# Supplementary material for: A UMLS-based spell checker for natural language processing in vaccine safety
Source: BMC Med Inform Decis Mak. 2007 Feb 12;7:3. doi: 10.1186/1472-6947-7-3 (PMC1805499; doi:10.1186/1472-6947-7-3)
Supplement: Additional file 13 — RAP application directory. Sets up directory for RAP (RDF application for PHP) source code [file 1472-6947-7-3-S13.gz › rap/doc/phpdoc/dataset/TriXParser.html]

xml version="1.0" encoding="iso-8859-1"?


Docs For Class TriXParser


## Class TriXParser

Description

Description |
Methods (details)

Temporary implementation of a TriX-Parser (Usable only with PHP > V5) Currently, it doesn't support any namespaces and has problems with typed literals.

So this parser only works with TRIX documents where the default namespace is the TRIX namespace.

History:

- access: public
- author: Daniel Westphal (http://www.d-westphal.de)
- version: V0.9

Located in /syntax/TriXParser.php (line 20)

Method Summary

Description |
Methods (details)

TriXParser
TriXParser
(GraphSet &$graphSet)

void
parseFile
(string $pathToFile)

void
parseString
(string $string)

Methods

Description |
Methods (details)

Constructor TriXParser (line 37)

Constructor Needs a reference to a graphSet

- access: public

TriXParser

TriXParser
(GraphSet &$graphSet)

- GraphSet
  &$graphSet

parseFile (line 59)

Parse from a file

- access: public

void

parseFile
(string $pathToFile)

- string
  $pathToFile

parseString (line 48)

Parse an XML string

- access: public

void

parseString
(string $string)

- string
  $string

Documentation generated on Thu, 7 Jul 2005 13:44:00 +0200 by phpDocumentor 1.3.0RC3
